# Understanding Subgenual Cingulate Functional Connectivity in Major Depressive Disorder Using 7T Fmri

**DOI:** 10.1192/bjo.2025.10192

**Published:** 2025-06-20

**Authors:** Laith Alexander, Mu Li, James Murrough, Laurel Morris

**Affiliations:** ^1^King’s College London, London, United Kingdom; ^2^South London and Maudsley NHS Foundation Trust, London, United Kingdom; ^3^Mount Sinai School of Medicine, New York, USA; ^4^Nuffield Department of Clinical Neurosciences, Oxford, United Kingdom

## Abstract

**Aims:** We aimed to determine differences in subgenual anterior cingulate cortex (sgACC) resting-state functional connectivity (rsFC) in Major Depressive Disorder (MDD) *vs.* healthy volunteers (HV) using 7-Tesla functional magnetic resonance imaging (fMRI). Abnormalities in the sgACC are linked to MDD, but the sgACC is anatomically and functionally diverse, including Brodmann area (BA) 25 (Cg25) and the subgenual portion of area 32 (Cg32). The differences in rsFC between Cg25 and Cg32 in MDD compared with HVs have not been directly examined. High-resolution 7T fMRI offers an unrivalled opportunity to measure differences in rsFC between these two subregions which otherwise suffer from signal dropout.

**Methods:** We used resting state 7T fMRI to compare rsFC between Cg25 and Cg32 in 40 patients with MDD, and 38 HVs. Within the MDD group, we correlated rsFC changes with anhedonia (SHAPS) and anxiety (STICSA) scores together with baseline high-sensitivity C-reactive protein (hsCRP) measures.

**Results:** Across all 78 participants, Cg25 and Cg32 showed regionally distinct rsFC patterns despite their proximity. Cg25 had increased rsFC to the orbitofrontal cortex, amygdala, hippocampus and dorsolateral (dl)PFC/BA46, while Cg32 showed increased rsFC to the perigenual (pg) and dorsal (d)ACC, dlPFC/BA9, posterior cingulate cortex (PCC), ventral striatum, and ventral tegmental area. When comparing MDD patients to HV, both Cg25 and Cg32 exhibited increased rsFC to the anterior (ant)PFC/BA10, amygdala and hypothalamus, together with key nodes of the default mode network (DMN), including pgACC, rostral ventromedial prefrontal cortex (vmPFC) and the PCC. rsFC to nodes of the central executive and salience networks, such as the right dlPFC/BA46 and the bilateral insula, was decreased. Within the MDD group, Cg32-antPFC/BA10 and Cg32-dlPFC/BA9 rsFC was positively correlated with anhedonia scores; additionally, subthreshold clusters were identified in the ventral striatum, pgACC and hypothalamus. Cg25-antPFC/BA10 and Cg25-PCC rsFC was negatively correlated with anxiety scores. Cg32 rsFC to the insula, dlPFC/BA9 and and dmPFC/BA10 showed negative correlations with hsCRP measures.

**Conclusion:** These findings suggest that sgACC subregions have distinct rsFC patterns which are altered in MDD. rsFC changes are differentially related to symptoms of anhedonia and anxiety, together with inflammatory status. This has important implications for the development of targeted neuromodulation treatment strategies.

